# A lipidomics-based method to eliminate negative urine culture in general population

**DOI:** 10.1128/jcm.00819-24

**Published:** 2024-09-16

**Authors:** Linda K. Nartey, Abanoub Mikhael, Helena Pětrošová, Victor Yuen, Pamela Kibsey, Mert Pekcan, Robert K. Ernst, Michael X. Chen, David R. Goodlett

**Affiliations:** 1 Department of Biochemistry and Microbiology, University of Victoria, Victoria, British Columbia, Canada; 2 Genome British Columbia proteomics center, University of Victoria, Victoria, British Columbia, Canada; 3 Vancouver Island Health Authority, Vancouver, British Columbia, Canada; 4 Department of Pathology and Laboratory Medicine, The University of British Columbia, Vancouver, British Columbia, Canada; 5 Faculty of Veterinary Medicine, Ankara University, Ankara, Turkey; 6 Department of Microbial Pathogenesis, University of Maryland, Baltimore, Maryland, USA; 7 Division of Medical Sciences, University of Victoria, Victoria, British Columbia, Canada; Johns Hopkins University, Baltimore, Maryland, USA

**Keywords:** urinary tract infection, uropathogens, microbial lipids, mass spectrometry

## Abstract

Urinary tract infections (UTIs) pose a significant challenge to human health. Accurate and timely detection remains pivotal for effective intervention. Current urine culture techniques, while essential, often encounter challenges where urinalysis yields positive results, but subsequent culture testing produces a negative result. This highlights potential discrepancies between the two methods and emphasizes the need for improved correlation in urinary tract infection (UTI) detection. Employing advanced lipidomics techniques, we deployed the fast lipid analysis technique (FLAT) on a clinical cohort suspected of having UTIs. Lipid fingerprinting by matrix-assisted laser desorption/ionization time-of-flight mass spectrometry (MALDI-TOF MS), directly from urine samples without *ex vivo* growth, correctly identified the common uropathogens within a 1 hour timeframe when compared to urine culture. FLAT analysis also identified urine samples without culturable pathogens (negative UTIs) with 99% microbial identification (ID) agreement, whereas urinalysis showed 37% ID agreement with the gold standard urine culture. In 402 urine samples suspected for UTI from outpatients, FLAT assay rapidly ruled out negative urines without the need for culture in 77% of all cases. The potential impact of this innovative lipidomic-based approach extends beyond conventional diagnostic limitations, offering new avenues for early detection and targeted management of urinary tract infections. This research marks a paradigm shift in urine culture methodology, paving the way for improved clinical outcomes and public health interventions.

**IMPORTANCE:**

This study employs a lipidomics-based method that promises to enhance the accuracy and reliability of urine culture diagnostics within 1 hour of sample collection. Our findings underscore the potential of lipidomics as a valuable tool in identifying and characterizing microbial populations present in urine samples and efficiently rule out negative urines, ultimately leading to improved patient care and management of urinary tract infections.

## INTRODUCTION

Urinary tract infections (UTIs) are one of the most common diseases worldwide, affecting approximately 150 million people every year, resulting in approximately 3.5 billion US dollars in healthcare costs (1). As a result, suspected UTIs are one of the most common presentations in a primary care setting. Although several urine biomarkers have been investigated for the diagnosis of UTIs, urine culture remains the gold standard (2). Approximately 80% of requested urine cultures come from outpatient settings, with as many as 70–80% of these samples showing no significant uropathogens (3). These negative urine cultures generate a considerable workload, which occupies microbiology laboratory staff leading to a waste of laboratory resources. Additionally, empiric antibiotic treatments may potentially have no clinical benefit and may increase the risk of adverse events, antimicrobial resistance, and healthcare costs.

Many patients with uncomplicated UTIs present clinically as straightforward cases that may not require additional testing beyond urinalysis. Because of the time-consuming nature of urine culture, many clinical laboratories have instituted reflexive workflows, such as urine culture, only after dipstick analysis has demonstrated positive findings. However, urine dipstick analysis for blood, nitrites, and leukocyte esterase can be prone to positive interferences leading to unnecessary urine culture investigations (4). Hence, it is clinically and operationally beneficial to develop a rapid alternative urine screening test in a culture-free manner with minimal human intervention.

Currently, matrix-assisted laser desorption/ionization time-of-flight (MALDI-TOF) mass spectrometry (MS) protein fingerprinting tests, such as the Bruker Biotyper, are standard microbial identification (ID) methods (5). These methods involve the use of MALDI-TOF MS to analyze the protein profiles of bacterial samples selected after culture. By comparing detected protein profiles to previously recorded protein profiles for various bacterial species in a library, bacteria can be identified. The protein-based methods mostly require microbial growth to produce a pure colony that often requires at least 18–48 hours (6). However, a study by Kitagawa et al. (7) investigated a modification to the standard method for bacterial identification that allowed directly from urine analysis. Their modified method involved addition of ultrasonication and centrifugation prior to placement of the sample on a MALDI plate. These modifications resulted in an improved identification accuracy direct from specimen over the standard method of 16.7–52.2% for Gram-positive cocci and 77.1–94.2% for Gram-negative rods (7). While identifications were improved, this and other related methods (8) have not seen widespread adoption potentially due to the complexities integrating the extra steps into a clinical workflow.

Here, we use a similar approach to identify bacteria by MALDI-TOF MS analysis, but instead of protein profiles, we use lipids (9). Complex and diverse lipids are a major component of bacterial membranes. In Gram-positive bacteria, the cell envelope consists of complex multi-layers of peptidoglycan enclosing a single membrane, a bilayer of lipids including cardiolipin (CL) and lipoteichoic acid (LTA) (10). In contrast, Gram-negative bacteria have a two-bilayer membrane with the inner membrane consisting of a single layer of peptidoglycan. Lipid A, the endotoxic portion of lipopolysaccharide (LPS), is embedded in the outer membrane of Gram-negative bacteria and exhibits species-specific structural diversity (10). These bacterial cell wall lipid components have been shown to provide a unique lipid barcode for the identification of individual microbial species in a manner similar to protein fingerprinting (9). These lipid barcodes for each bacteria represent signature ions allowing individual bacteria to be uniquely identified. The microbial lipid biomarkers used in this study consist mainly of cardiolipin and lipid A.

A recent study by Sorenson et al. (11) presented a method for direct lipid extraction on a MALDI plate, referred to as fast lipid analysis technique (FLAT). Similar to the chemistry used in traditional lipid microextraction, FLAT differs mainly in extraction of the aforementioned microbial lipids directly onto a MALDI plate. Thus, the FLAT assay reduces the effort needed to observe the lipids that serve as barcodes to identify bacteria and fungi, as well as detect membrane-based antimicrobial resistance (9, 12–14). The FLAT method simplifies sample preparation, allowing rapid analysis direct from the sample with only 1 µL urine. This simplified direct from specimen workflow allows FLAT to be integrated seamlessly into existing laboratory processes because the FLAT method is nominally identical with the protein-based methods from the time the sample is placed on the MALDI plate. One impediment to use of protein-based methods for direct from specimen analysis is their higher molecular weight that produces mass spectra that are generally more complicated than lower molecular weight lipids. Additionally, any added complexity from host cellular proteins makes it even more challenging to accurately identify proteins using MALDI MS (15). In contrast, lipids are smaller and typically produce simpler mass spectra with fewer ions, making analysis more straightforward.

Given that FLAT circumvents the need for culture, results can be ready within an hour of the laboratory receiving a sample. Previously, Yang et al. have shown that microbial lipid profiles can accurately identify microbial colonies directly from known Gram-negative urine samples via FLAT with MALDI-TOF MS (12). However, their study only focused on analysis of known Gram-negative UTI urine samples.

Following the work of Yang et al., we used lipid biomarkers analyzed by MALDI-TOF MS to determine FLAT's ability to discern positive from negative urines in a large clinical cohort. Over 400 suspected UTI samples from outpatient settings, excluding pregnant women and patients with sexually transmitted disease, were used for this study. The overall aim of this study was to evaluate the use of FLAT to eliminate the need for urine culture in the general population.

## MATERIALS AND METHODS

### Sample collection

In this study, 435 urine specimens were collected from outpatients suspected to have UTIs from Victoria General and Royal Jubilee Hospital, British Columbia. Maternity care and sexually transmitted infection (STI) clinics were excluded. Urine samples were requested by clinicians as part of urinary tract infection investigations, either by (1) urinalysis (dipstick; Roche Diagnostics, 11063 Stn CV, Montreal, QC) and urine culture or (2) urinalysis with reflex to urine culture. The criteria for reflex to urine culture were established based on positive urinalysis results incorporating any single or combined indications, including positive hemoglobin (Hb), nitrites, and leukocyte esterase. Negative urine analysis without urine culture was excluded. Bacterial isolates from urine cultures were identified using the protein Biotyper (MBT Sirius; Bruker, Bremen, Germany). Based on the inclusion criteria, all urine samples were collected from morning midstream clean-catch urine, without considering age and gender of the patients. Urine culture was not performed on 33 of the 435 (7.5%) samples that did not pass inclusion criteria.

The remaining 402 urine samples were tested by urinalysis and urine culture as part of the VIHA standard of care. The urine samples were obtained in sterile BD Vacutainer urine C&S Preservative Tubes (BD, Franklin Lakes, NJ, USA). Specifically, a 1 mL aliquot was allocated from each sample for FLAT analysis within 3 days of receipt of the UTI sample. FLAT results were compared with corresponding urinalysis and urine culture reports. In our study, we used a colony count threshold of ≥10^5^ CFU/mL (colony-forming units per milliliter) to classify a culture as positive. This threshold aligns with standard clinical microbiology practices, particularly for urinary tract infections (UTIs). Conversely, a culture is considered negative if it is reported as “light or mixed growth (0.1–0.9 × 10^5^ CFU/mL),” “no growth (<0.1–0.9 × 10^5^ CFU/mL),” and “no uropathogens isolated” (Table S1). The VIHA microbiology laboratory does not perform further UTI investigations in urine cultures reported as light or mixed growth since this may or may not indicate an infection, depending on the clinical context. Hence, all such reported outcomes were classified as negative urine cultures in this study.

**TABLE 1:** Patient demographics (*n* = 402)

	Female	Male	Gender X
Children (1–12 years)	10	4	
Adolescents (13–17 years)	2	4	
Adults (18 years or older)	110	59	1
Older adults (>65 years)	103	109	

**TABLE 2:** Positivity of urine culture and urinalysis in the study cohort

*n* = 402	Positive	Negative
Culture	88 (22%)	314 (78%)
Urinalysis	285 (71%)	117 (29%)

### FLAT analysis

Lipid A mass spectral analyses were performed using the FLAT assay described by Sorensen et al. (11). A 1 mL aliquot of each urine was transferred into an Eppendorf tube and analyzed using FLAT.

For this study, stainless-steel, 96-well, disposable MALDI target plates (MFX μFocus plate 12 × 8c 2,600  µm 0.7 T; Hudson Surface Technology, Inc., South Korea) were used. Urine samples were processed in two ways. First, 1 µL of urine was spotted directly onto the MALDI target plate, and second, 1 mL of urine was centrifuged at 8,000 g for 5 min to generate a urine pellet, after which 1 µL bacterial pellet was spotted onto a MALDI target plate. Next, 1 µL of a citric acid buffer containing 0.2 M anhydrous citric acid and 0.1 M trisodium citrate dihydrate (Fisher Chemical) was placed on top of each dried sample. The MALDI target plate was then incubated at 110°C for 30 min in a humidiﬁed chamber and gently rinsed with endotoxin-free water. Each sample was spotted on the MALDI plate in triplicate. Subsequently, 1 µL norharmane (10 mg/mL) matrix (Sigma Aldrich) dissolved in 2:1  vol/vol MS-grade chloroform and methanol (both from Fisher Chemical) was spotted onto the extracted lipid sample on a MALDI target plate, allowed to dry and then analyzed by MALDI-TOF MS.

### Modifications to FLAT

Cloudy samples were diluted in 10-fold prior to FLAT analysis. Briefly, 100 µL of cloudy urine was diluted in 900 µL sterile deionized water. Next, 1 µL of diluted urine was spotted directly onto the MALDI target and analyzed by FLAT assay described above.

After the initial processing steps described above, a second cohort of 70 urines suspected to contain Gram-positive bacteria underwent an additional sonication step to improve the detection sensitivity of FLAT. This step involved subjecting the urine samples to sonication for 10 min before proceeding with the FLAT analysis protocol as outlined. Briefly, 1 mL of urine was sonicated for 10 min, and 1 µL of urine was spotted directly onto the MALDI target plate and then centrifuged at 8,000 g for 5 min to generate a urine pellet, after which 1 µL bacterial pellet was spotted onto a MALDI target plate. Acidified and incubated in a humidified chamber at 110°C for 30 min. The plate was rinsed and 1 µL of norharmane matrix was added to each spot. Samples were then analyzed by MALDI-TOF MS.

### MALDI-TOF MS analysis and pathogen identification

MALDI-TOF MS analysis was conducted using a Bruker Microflex (Bruker, Bremen, Germany) in negative ion and linear mode. Analyses were conducted at 65% global intensity with 500 laser shots for each acquired mass spectrum. Mass spectra were collected between 1000 *m/z* and 2,400 *m/z*. Acquired mass spectral data were processed using flexAnalysis (v3.4) software with smoothed and baseline corrections using monophosphoryl lipid A (MPLA) as a post-acquisition mass calibrant. Samples are called positive when the signal to noise is >10, relative intensity threshold of 1%, peak height and width of *m/z* ± 2.5 and 80%, respectively. After MALDI-TOF MS analysis, pathogens were identified by comparing sample mass spectra to a developed microbial library (SI 1) and previously acquired reference spectra (9, 11, 12, 16). In routine standard of care, cultures were identified using the protein Biotyper (MBT Sirius; Bruker, Bremen, Germany) and the 3.4.207.48./BDAL 11 organism library.

### Identification of non-pathogenic markers by FLAT

Analyses of molecules of unknown structure at specific *m/z* values, some of which interfered with known microbial lipid signature ions, were analyzed using FLAT*^n^* (14), the tandem-MS version of FLAT on a Bruker MALDI trapped ion mobility spectrometry (TIMS) TOF MS (Bruker, Bremen, Germany). The instrument was calibrated before every experiment by direct infusion of electrospray tuning mix (Agilent, Santa Clara, CA, USA). To acquire tandem mass spectra, the precursor ion *m/z* value of interest was entered to the third decimal point with isolation width and collision energy set to 4 *m/z* and 100–110 eV, respectively. Mass spectra were acquired in negative ion mode with a mass resolution of 60,000 at *m/z* 400. After manual analysis of the tandem mass spectra of unknown ions, structures were hypothesized using mMass (v5.5.0) software and subsequently confirmed by manual analysis in ChemDraw v18.0 (PerkinElmer informatics, Waltham, MA, USA).

### Analysis of known Gram-positive cohort

The FLAT assay was performed on a second cohort of 70 known Gram-positive urine samples with slight modification. Briefly, 1 mL urine was sonicated for 10 min before analyzing via FLAT on both direct urine and pellets from this modification.

### Data analysis

Specificity and sensitivity of the results were calculated using the following formulas: SE = TP/(TP + FN) ×100% and SP = TN/(TN + FP) × 100% where the positive predictive value (PPV) and negative predictive value (NPV) were calculated as follows: PPV = TP/P; NPV = TN/N where FN = false negative; FP = false positive; N = negative; P = positive; SE = sensitivity; SP = specificity; TN = true negative; and TP = true positive). Mass spectral data were analyzed using flexAnalysis (v3.4) software processed with smoothed and baseline corrections.

## RESULTS

**Fig 1:**
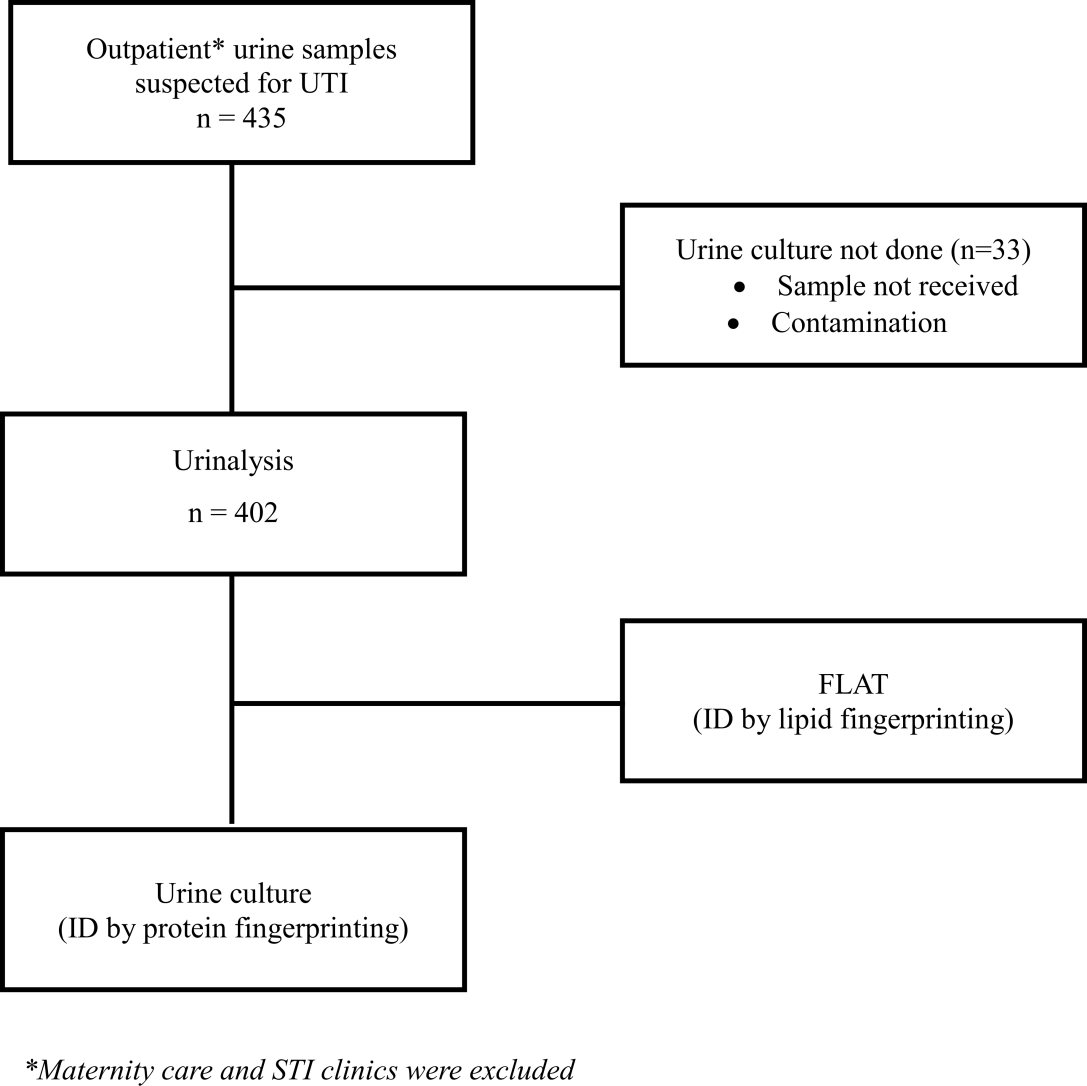
Study cohort inclusion/exclusion criteria.

**Fig 2:**
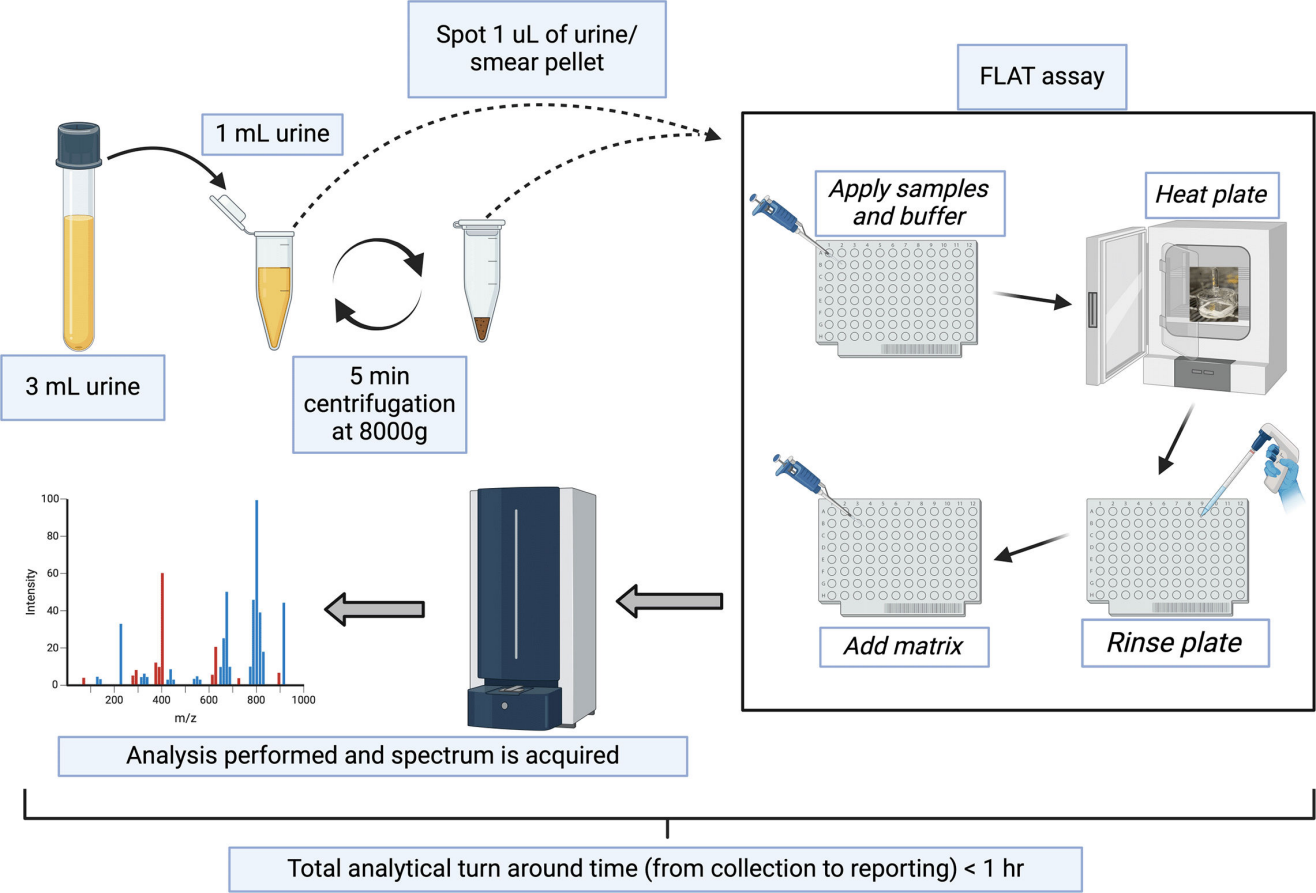
Experimental workflow for direct-from-urine analysis. Figure created with BioRender.com.

**Fig 3:**
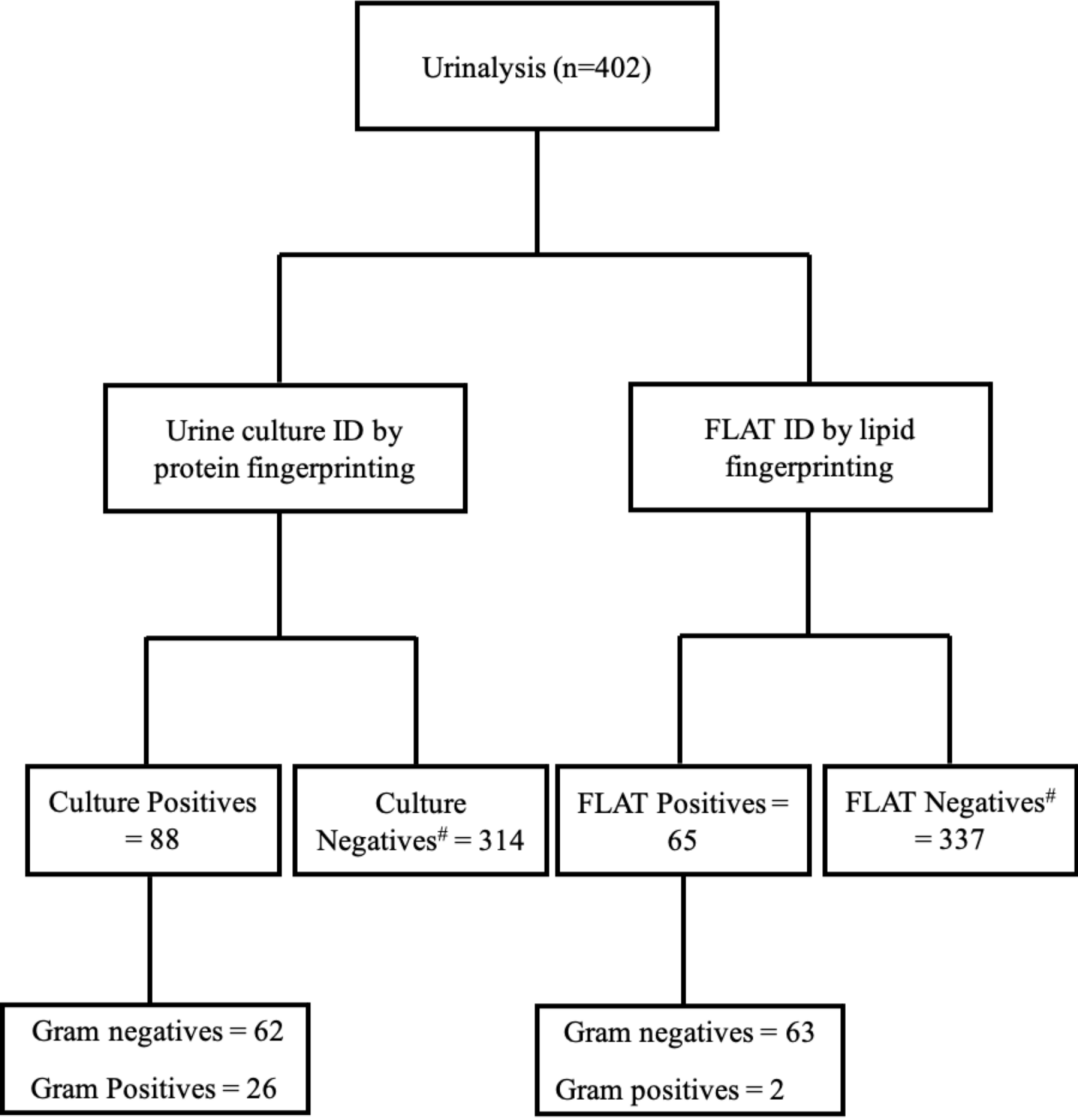
Comparison of pathogen identification by proteins and lipids. #Samples are classified as negative when 0.1–0.9 × 10^5^ CFU/mL.

A total of 435 samples were collected for this study (Fig. 1 and 2). Of those, 33 coming from maternity care or STI clinics were excluded leaving 402 urine samples for analysis. The age of the study population ranged from 15 months to 98 years. Out of the 402 samples, 95% were aged >18 years old (42% were >65), 56% were female, and one patient was identified as gender X (Table 1). Overall, urinalysis reported 71% and 29% of the samples as positive and negative, respectively; whereas urine culture reported 22% and 78% of the samples as positive and negative (Table 2; Fig. 3). The majority of positive urine cultures showed Gram-negative uropathogens (78%), with *Escherichia coli* being the most common at 78% (Table 2).

### Modifications to FLAT

Approximately ~3% of the 402 samples were observed to be cloudy containing sediment, resulting in mass spectra with higher noise levels than those without sediment. “Noise” in this case refers to ions that are not of microbial origin. These mass spectra with higher background noise relative to the signal intensity of the signature and satellite ions make interpretation slightly more difficult. A higher level noise can obscure the signature ions representative of individual microbes leading to mass spectra with poorer signal-to-noise (S/N) ratio. To overcome this problem, these cloudy samples were diluted 10-fold, after which negative samples produced less noisy MALDI-MS spectra whereas positive samples (0.7%) produced mass spectra with high signal-to-noise (S/N) ratio of bacterial lipid ions. Furthermore, only 8% of the 402 samples known to be Gram positive were detected by FLAT. To improve detection of Gram positives, sonication prior to FLAT was incorporated. In an analysis of a second cohort of 70 known Gram-positive samples, this improved detection of Gram positives to 51%.

### Identification of uropathogens by FLAT

**Fig 4:**
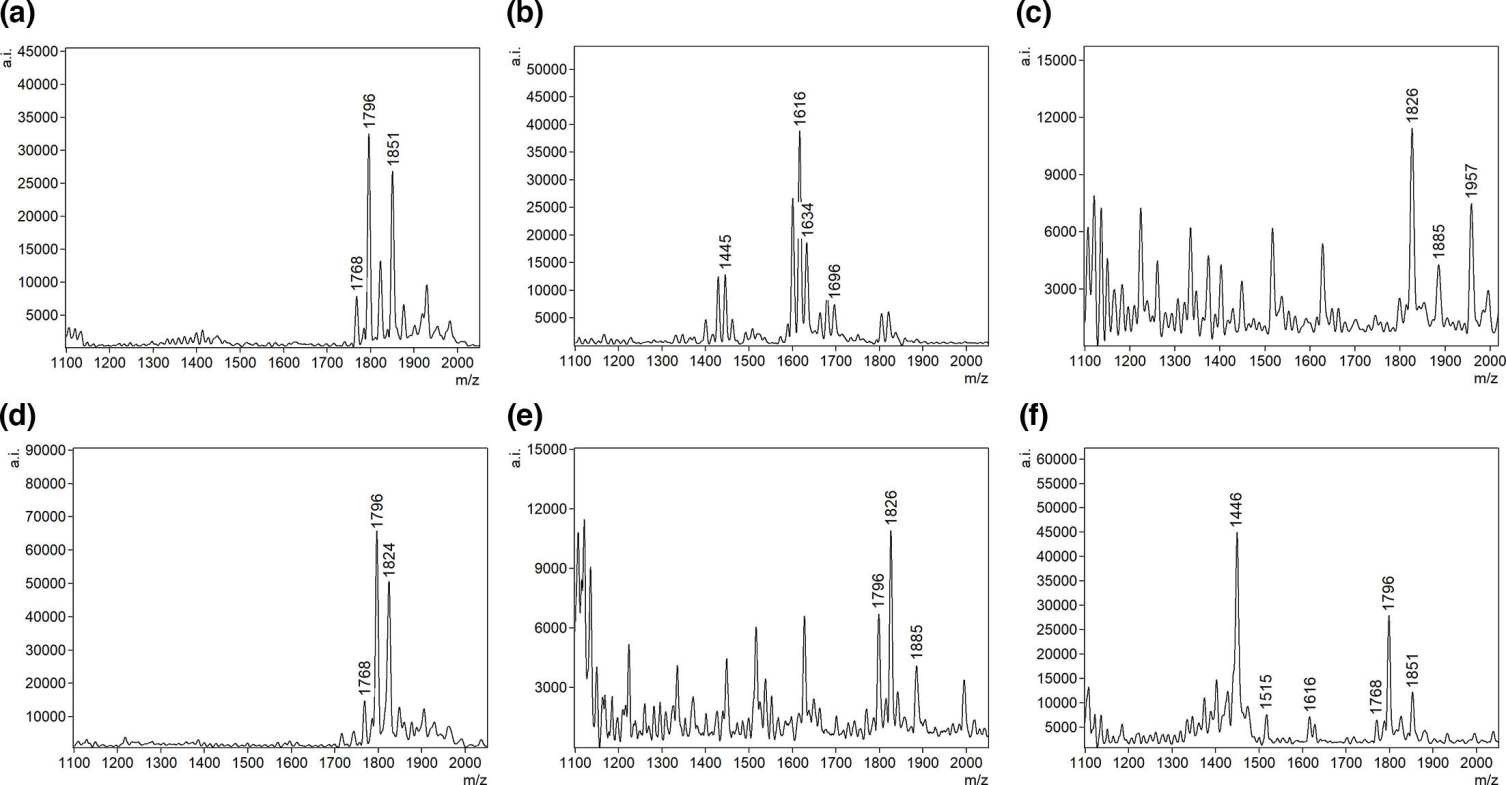
Representative FLAT mass spectra of detected Gram-negative uropathogens. (a) *E. coli,* (b) *P. aeruginosa,* (c) *Proteus* sp., (d) *Enterobacter* sp.*,* (e) *Klebsiella* sp., and (f) polymicrobial (*P. aeruginosa* and *E. coli*).

**Fig 5:**
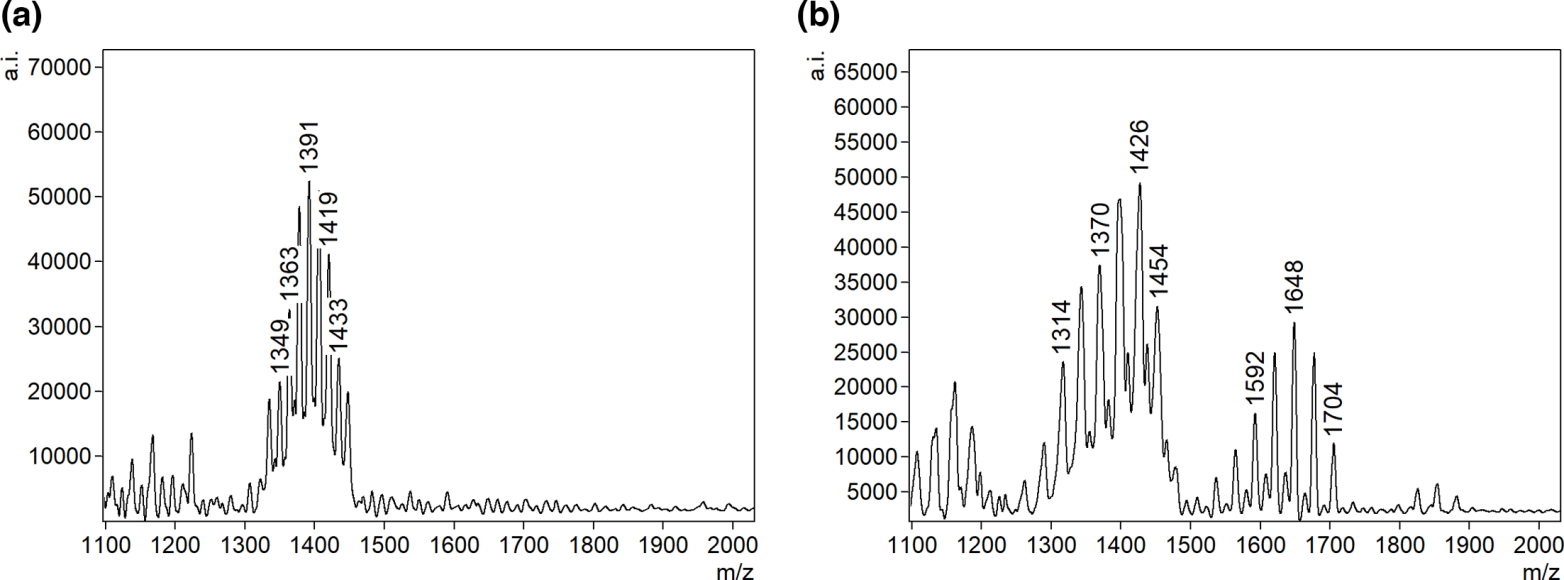
Representative FLAT mass spectra of detected Gram-positive uropathogens. (**a**) *S. epidermidis* and (**b**) *A. urinae*.

Directly from 1 µL of urine, we identified common uropathogens including 51 *Escherichia coli* (78%), six *Klebsiella* spp (9%)*,* one *Pseudomonas aeruginosa* (2%)*,* three *Enterobacter cloacae* (5%)*,* three *Proteus mirabilis* (5%), and one sample that was polymicrobial containing *P. aeruginosa* and *E. coli* (Fig. 4). Two Gram-positive bacteria, *S. epidermidis* and *A. urinae*, were also detected (Fig. 5).

### Method comparison

The FLAT and culture results showed 93% concordance (Table 3). Overall, the FLAT assay had a sensitivity of 70% and specificity of 99% with positive and negative predictive values of 93% and 99%, respectively (Table 3). Table 3 also indicates three false positives where FLAT identified a positive result, but the culture was negative. This discrepancy arises because our criterion for culture positivity was a threshold of ≥10^5^ CFU/mL. Cases of light growth (10^3^–10^5^ CFU/mL) and mixed growth were often classified as negative if they did not meet this threshold. From our limit of detection (LoD) study, Fig. S2 to S4, FLAT detects pathogens even at 10^3^ CFU. For negative urine cultures, the FLAT assay was 99% in concordance whereas urinalysis was 37%. (Table 4). Additionally, the FLAT assay was 97% (*n* = 88) and 8% (*n* = 26) in ID agreement with Gram-negative and Gram-positive cultures, respectively (Table 5). An additional 70 known Gram-positive urines were obtained to increase the sample size. After adding a 10 min sonication step prior to FLAT, Gram-positive bacterial detection increased from 8% of the initial cohort of 402 to 51% of the new cohort of 70 samples (Table 6).

**TABLE 3:** A 2 × 2 diagnostic table of FLAT assay

		UTI results		
		Culture +	Culture −	
FLAT results	Positive	True positive (62)	False positive^*a*^ (3)	Positive predictive value = 95%
	Negative	False negative (26)	True negative (311)	Negative predictive value = 92%
		Sensitivity = 70%	Specificity = 99%	

^*a*^ False positives (*n* = 3) are defined as cases where FLAT indicated a positive result, but culture results were negative based on a threshold of ≥10^5^ CFU/mL.

**TABLE 4:** Method comparison results of FLAT, urinalysis, and culture of negative urines

Culture neg	Urinalysis (dipstick) neg	Flat neg	Comments
*N* = 314	117 (37%)	311 (99%)	FLAT oversensitive (three false positives by FLAT analysis)

**TABLE 5:** Method comparison results of FLAT, urinalysis, and culture of positive urines

Culture pos	Urinalysis (dipstick) pos	Flat pos	Comments
Gram – (*n* = 62)	62 (100%)	60 (97%)	Two undetected bacteria by FLAT (*Acinetobacter* and *Citrobacter* spp.)
Gram + (*n* = 26)	24 (92%)	2 (8%)	

**TABLE 6:** Detection of known Gram-positive bacteria by FLAT

Known Gram-positives		
Microbe	FLAT detected	Sample received
*Enterococcus faecalis* not VRE	13	26
*Enterococcus faecium* VRE	6	6
*Streptococcus agalactiae* group B	7	10
*Aerococcus urinae*	2	2
*Staphylococcus aureus*	2	6
*Staphylococcus epidermidis*	1	4
*Enterococcus faecium*	1	7
*Staphylococcus saprophyticus*	1	4
*Streptococcus pyogenes*	0	2
*Staphylococcus capitis*	2	2
*Staphylococcus lugdunensis*	1	1
Total	36 (51%)	70

### Identification of potential non-microbial interferences by FLAT

**Fig 6:**
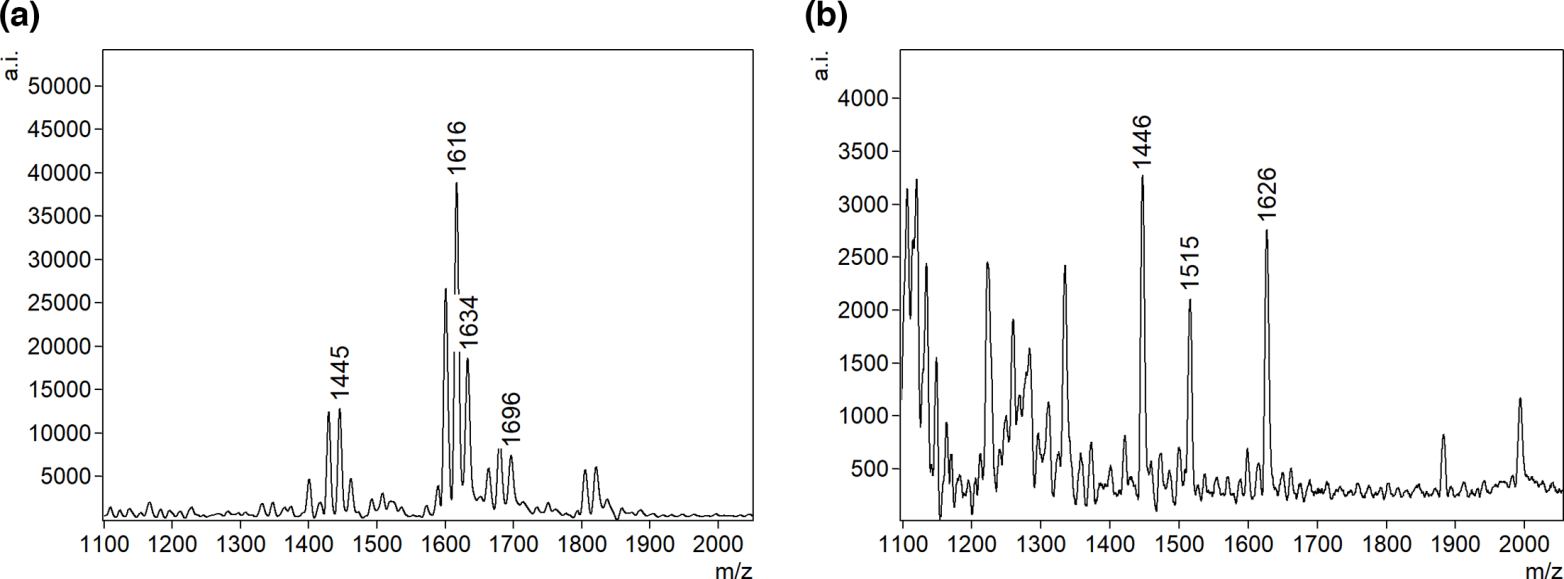
FLAT mass spectra showing *m/z* 1,447–1,449 interference. (**a**) Lipid A mass spectra of *P. aeruginosa* and (**b**) host cardiolipin.

**Fig 7:**
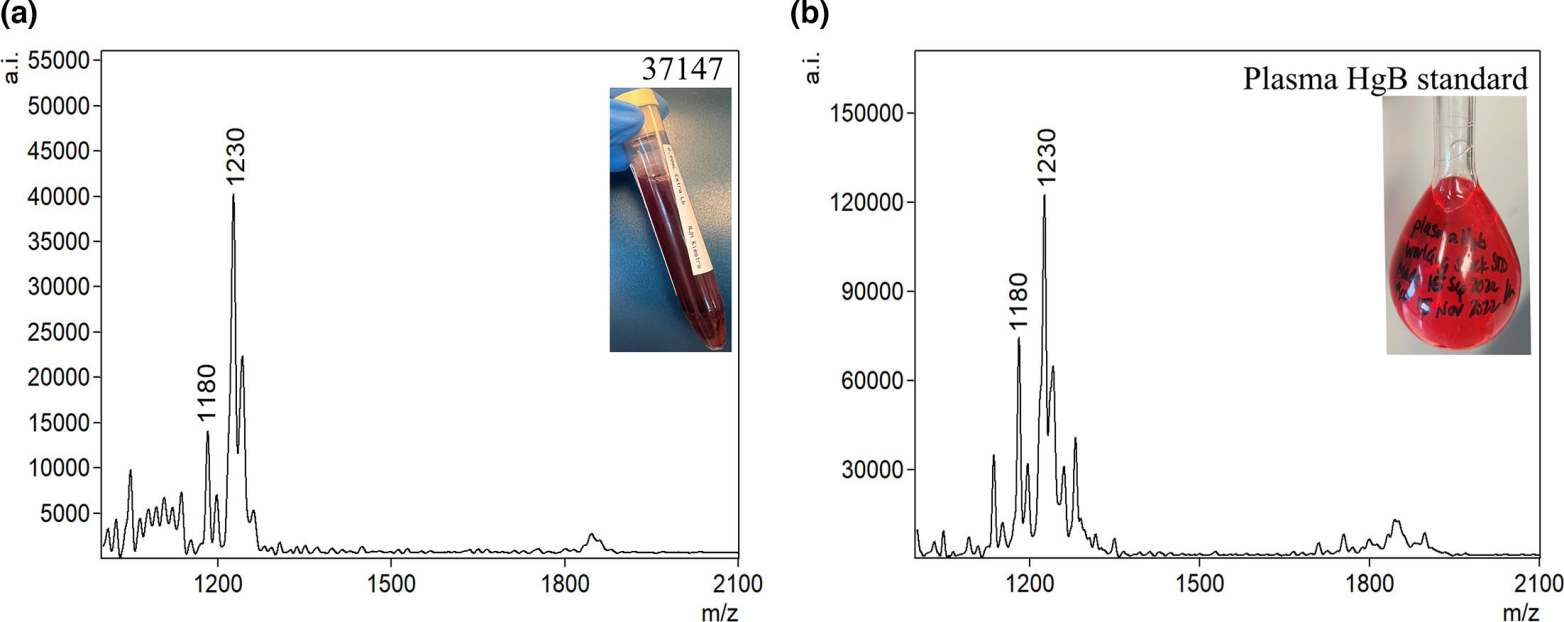
FLAT mass spectra of heme: (**a**) patient urine with blood and (**b**) plasma HgB standard.

In addition to microbial lipids, FLAT detected host cardiolipins and heme degradation products. Approximately 40% of urine samples showed a cluster of ions at *m/z* 1,447–1,449, which is similar to one of the signature ion of *P. aeruginosa* lipid A (Fig. 4b). However, samples containing these ions of *m/z* ~ 1,447–1,449 by FLAT failed to produce viable colonies when isolated on a culture media (Fig. 6b). Tandem MS results of the *m/z* 1,448, ion cluster showed similar fragmentation to a mammalian cardiolipin reported by Kim et al. (17) (Fig. S5). Additionally, an ion at *m/z* 1,230, which was present in all urine samples with blood (6.5% of initial cohort of 402) (Fig. 7a), was detected by FLAT. After FLAT analysis on a plasma Hb standard obtained from the clinical laboratory, we observed the presence of a similar ion at *m/z* 1,230 (Fig. 7b). Manual analysis of the tandem MS spectrum of this ion compared against the same *m/z* value from the plasma standard predicted this ion to be a heme dimer (Fig. S6).

## DISCUSSION

The culture of urine samples remains the gold standard for diagnosing UTIs, but this process takes 24–72 hours to complete, and inappropriate sampling or storage conditions can lead to false positive results (18). A rapid and inexpensive test with high negative predictive value would reduce unnecessary urine culture and inappropriate empiric antimicrobial treatment. In this study, we present FLAT, a lipid-based method that allows direct lipid extraction on a MALDI plate for pathogen detection in under an hour from receipt of a sample. We investigated the diagnostic performance of FLAT in a clinical cohort to highlight the potential benefits of this assay in comparison with conventional urine culture.

FLAT analysis identified urine samples without culturable pathogens (negative UTIs) with 99% ID agreement, whereas urinalysis showed 37% ID agreement with the gold standard urine culture. This technique shows that microbial membrane lipids analyzed by MS represent biomarkers that can be used to identify monomicrobial or polymicrobial specimens directly from urine samples without the need for culture. The FLAT assay identified common uropathogens directly from urine samples within 1 hour of collection. In 402 urine samples suspected for UTI from outpatients, FLAT assay rapidly ruled out negative urines without the need for culture in 77% of all cases.

It is estimated that over 80% of UTIs are caused by Gram-negative bacteria and predominantly by *E. coli* (19). From our study, the FLAT data showed an overall sensitivity of 70% and a specificity of 99% with positive and negative predictive values of 95% and 92%, respectively, for both Gram-positive and Gram-negative bacteria. The low sensitivity is the result of inconsistent detection of Gram-positive bacteria, which might be due to relatively lower amounts of cardiolipin signature ions being released from Gram positives than lipid A from Gram negatives in these urine samples (20, 21). Additionally, the FLAT extraction process may be less effective in disrupting Gram-positive cell walls. Given that FLAT was developed to isolate lipid A from Gram-negative bacteria, it is likely that the rigidity of the Gram-positive cell wall prevents the efficient release of cardiolipin from Gram-positive bacteria. This seems likely given that lipids in general have good ionization efficiency (22) and is bolstered by the report of Angelini et al. who showed the use of cardiolipin fingerprinting by MALDI-TOF as a screening tool with no difficulties in ionization (23). The LoD studies provided in Fig. S2 to S4 demonstrate organism-specific differences. For instance, *E. coli* showed a lower LoD compared to *E. faecalis*, indicating higher sensitivity for certain bacterial strains.

To improve the sensitivity of FLAT, we carried out centrifugation of urine samples prior to analysis in an attempt to enrich pathogens. Gram-negatives showed an improved sensitivity of 94% and specificity of 99% with positive and negative predictive values of 95% and 99%, respectively. In contrast, centrifugation did not improve detection of Gram-positive bacteria (15). To overcome this diagnostic limitation, we added a 10 min sonication step to sample preparation. It was also noted that less than 7% of samples tested positive for Gram-positive bacteria in our cohort of 402 samples. Subsequently, we obtained another 70 urine samples from the microbiology laboratory with confirmed growth of Gram-positive bacteria. Improved cardiolipin detection was observed after sonication with an increased sensitivity for Gram-positive bacteria to 51%. We believe that this increased cell wall disruption allowed for a more comprehensive release of the cardiolipins leading to a higher detection rate. This implies that, had sonication been used in the original cohort, sensitivity of FLAT would have been about 85% due to increased detection of Gram-positive containing samples. Encouraged by this outcome, we are continuing method development to further improve Gram-positive detection. It is imperative to note that this extra 10 min sonication step does not affect Gram-negative detection. Hence, a 10 min sonication is recommended for every urine samples in order to detect both Gram-negatives and Gram-positives with high confidence.

The mass range of bacterial cardiolipin and lipid A is between *m/z* 1,000 and 2,400. In some cases, there were interfering ions present that initially led to false positives. To prevent such false positive identifications, it was essential to define non-microbial ions present in this mass range of interest that were present at similar *m/z* values to the signature ions (Table S2). Urine, being a by-product of metabolism, can contain non-microbial chemical components that might interfere with FLAT identification of pathogens via their signature ions. For example, in this study, approximately 40% of the samples diagnosed as negative for bacteria showed an ion at *m/z* 1,447–1,449, which is similar to the reported as one of the signature ion for *P. aeruginosa* lipid A (9). However, tandem MS results of *m/z* 1,448 showed similar fragments to what Kim et al. (17) reported as a mammalian cardiolipin. We note that this putative cardiolipin was found in suspected UTI samples that were both positive and negative for bacteria and were not gender or age specific.

In all urine samples positive for Hb, FLAT detected an ion at *m/z* 1,230. With hematuria being one of the symptoms of UTIs, it was paramount to identify and characterize this ion. Tandem MS results of this ion compared against a plasma standard analyzed by FLAT confirmed that it was, in fact, a heme dimer. In 1995, Benesch and Kwong reported that hemoglobin can easily be transferred between body fluids in the form of dimers (24). This phenomenon explains why MS did not detect heme *m/z* 614 but rather showed a heme dimer at *m/z* 1,230.

There was one case of polymicrobial infection that was accurately identified by FLAT as *E. coli* and *P. aeruginosa* with distinct ions and their respective *m/z* values were detected without interference. The ability to detect polymicrobial infections directly from urine within 1 hour will guide appropriate antibiotic therapy and improve clinical outcome (25). However, to further make the point that FLAT can readily detect individual microbes in polymicrobial samples, we produced a contrived example where we show that FLAT can detect all three organisms after mixing equal parts of *E. coli, P. aeruginosa*, and *S. aureus* (Fig. S1). Theoretically, this ability is related to the resolving power of the mass spectrometer. In the case of protein mass spectra, the width of ions is much broader than that for lipid mass spectra: roughly 10–20 versus 1–2 *m/z*. This difference means that there are fewer chances for signature ions of individual microbes to overlap in a lipid extract of multiple organisms than would be the case for their comparable protein ions. With regard to FLAT, overlapping ions will only occur when organisms are from the same family. This is evident in the Enterobacteriaceae family where the signature ions (base peak) for lipid A mass of *E. coli* and *Enterobacter* sp all occur at 1,796, but their satellite ions (ions that always show up in addition to the base peak) differ slightly, providing some ability to distinguish one from the other.

The automation and availability of artificial intelligence (AI)-assisted screening for negative cultures in clinical laboratories is emerging as a promising area of focus. This innovation has the potential to improved efficiency in the microbiology laboratory. Our FLAT assay has the capacity for batch analysis of up to 92 samples per MALDI plate with an analytical turnaround time of <1 hour, and it eliminates the need for culture in the low-risk general population. Our future research direction includes interpretation of lipid spectra and pathogen identification by a software solution that can be automated for post-analytical result distribution.

### Conclusion

The ability to identify pathogens without need for culture can allow for faster pathogen identification, reduced time to appropriate antimicrobial therapy, reduced use of disposable plasticware, and improved patient outcomes. Rapid exclusion of urinary tract infections and the elimination of culture-negative urines in low-risk population avoid unnecessary empiric antibiotic treatment and save healthcare resources. When compared to the current standard of care, FLAT demonstrated accurate microbial identifications of Gram-negative bacteria with an analytical turnaround time of <1 hour. The FLAT assay provides a rapid alternative urine screening test that out-performed urinalysis (dipstick) with lower false positive rate. Notably, FLAT was able to rule out UTI with 99% accuracy. Finally, we suggest that use of FLAT as a screening test for suspected UTIs could eliminate ~77% of negative urine cultures. It is important to underscore that FLAT offers significant advantages in terms of rapid turnaround time, which can expedite the initiation of appropriate antimicrobial therapy and improve patient outcomes. The lipid-based method is more cost-effective due to its reduced reagent and consumable requirements. The FLAT workflow makes the lipid-based method highly adoptable in routine clinical laboratories, hence well-suited for high-throughput environment.

Uropathogens identified by FLAT will still require confirmation by urine culture for bacterial speciation and antibiotic susceptibility testing.

## Data Availability

Data supporting the findings of this study are available upon request.

## SUPPLEMENTAL MATERIAL

Supplemental material jcm.00819-24-s0001.pdf Tables S1 and S2; Fig. S1 to S6.

ASM does not own the copyrights to Supplemental Material that may be linked to, or accessed through, an article. The authors have granted ASM a non-exclusive, world-wide license to publish the Supplemental Material files. Please contact the corresponding author directly for reuse.
