# Supplementary material for: A lipidomics-based method to eliminate negative urine culture in general population
Source: J Clin Microbiol. 2024 Sep 16;62(10):e00819-24. doi: 10.1128/jcm.00819-24 (PMC11481538; doi:10.1128/jcm.00819-24)
Supplement: Supplemental material — Tables S1 and S2; Fig. S1 to S6. [file jcm.00819-24-s0001.pdf]

SUPPLEMENTAL INFORMATION (SI)

SI. Table 1: Clinical definitions and terminologies

<table>
<tr><th>Results</th><th colspan="2">Report</th></tr>
<tr><td>Growth</td><td colspan="2">Reported colony count interpretation is based on number of colonies</td></tr>
<tr><td>No Growth</td><td colspan="2">No growth (<0.1-0.9 x 10^5 CFU/mL)</td></tr>
<tr><td rowspan="4">Light or mixed growth</td><td colspan="2">If no AST performed and no organism name is being reported
Reported colony count interpretation is based on number of colonies:</td></tr>
<tr><td>If actual colony count is:</td><td>Then reported as:</td></tr>
<tr><td>1-9 colonies</td><td>0.1-0.9 x 10^5 CFU/mL</td></tr>
<tr><td>10-99 colonies</td><td>1-9 x 10^5 CFU/mL</td></tr>
</table>

SI. Table 2: Microbial library of common uropathogens from study cohort. These were isolates obtained from Vancouver Island Health Authority (VIHA).

**Microbe**	**Signature Ion (*m/z*)**	**Satellite Ions (*m/z*)**
Acinetobacter baumannii	1728	1700, 1883, 1911
Citrobacter koseri	1797/1825	1905, 1958, 2035, 2063
Enterobacter aerogenes	1797	1859, 1877, 1929, 2036
Enterobacter cloacae	1796	1852, 1877, 1905, 2045
Escherichia coli	1796	1876, 1964
Enterococcus faecalis	1430	1402,1416, 1456
Hafnia alvei	1797	1877, 1928, 2035, 2166
Klebsiella pneumoniae	1825	1797, 1888, 1905, 2063
Klebsiella oxytoca grp	1825	1767, 1888

Klebsiella variicola	1825	1614, 1887, 1905, 2079
Pseudomonas aeruginosa	1446	1616
Pantoea agglomerans	1797	1877, 1931, 2045, 2099
Providencia rettgeri	1956	1825, 2063, 2124, 2194
Staphylococcus aureus	1351	1295, 1323, 1379, 1407
Serratia marcescens	1857	1797,1825,1905,1929, 2063
Proteus vulgaris	1826	1729, 1957, 2062, 2195
Proteus mirabilis	1826	1729, 1956, 2063, 2194
Aerococcus urinae	1430	1327, 1403
Morganella morganii	1376/1797	1928, 2035
Enterococcus faecium	1327	1313, 1375, 1403
Staphylococcus saprophyticus	1323	1295, 1351,1387, 1414
Candida albicans	1356	1328, 1335, 1349,1692
Enterococcus avium	1389	1347, 1418
Streptococcus agalactiae	1403	1375, 1431

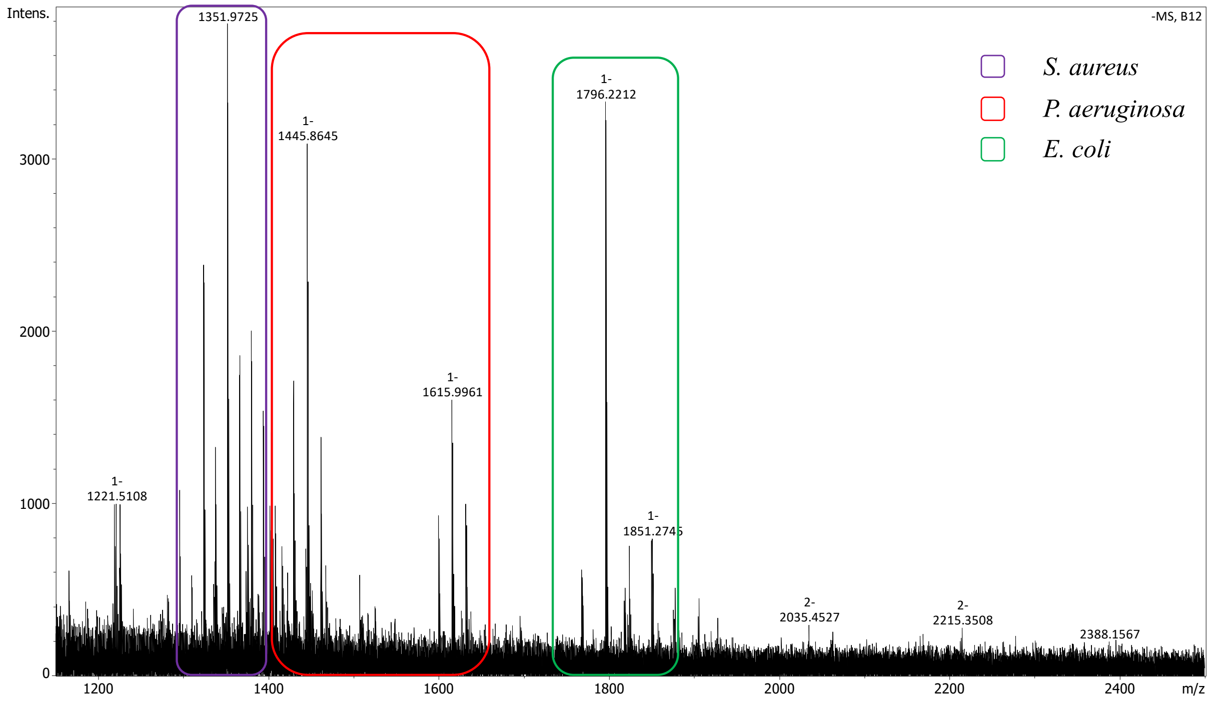

SI. Fig 1: Polymicrobial urine sample. UTI positive sample containing *E.coli*, *P. aeruginosa* and *S. aureus*.

Limit of Detection study

This study involves systematically diluting known concentrations of bacterial cultures to assess the lowest concentration at which FLAT can reliably detect and identify pathogens. Samples were analyzed in triplicate.

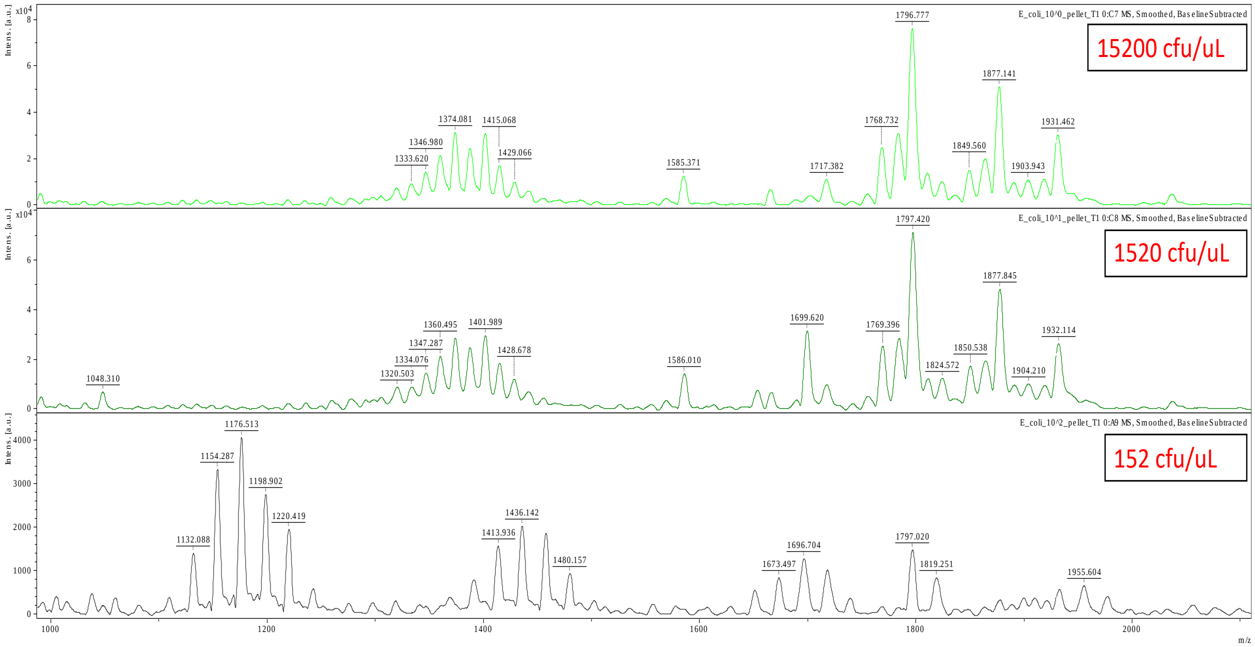

SI. Fig 2: Limit of detection of *E. coli*

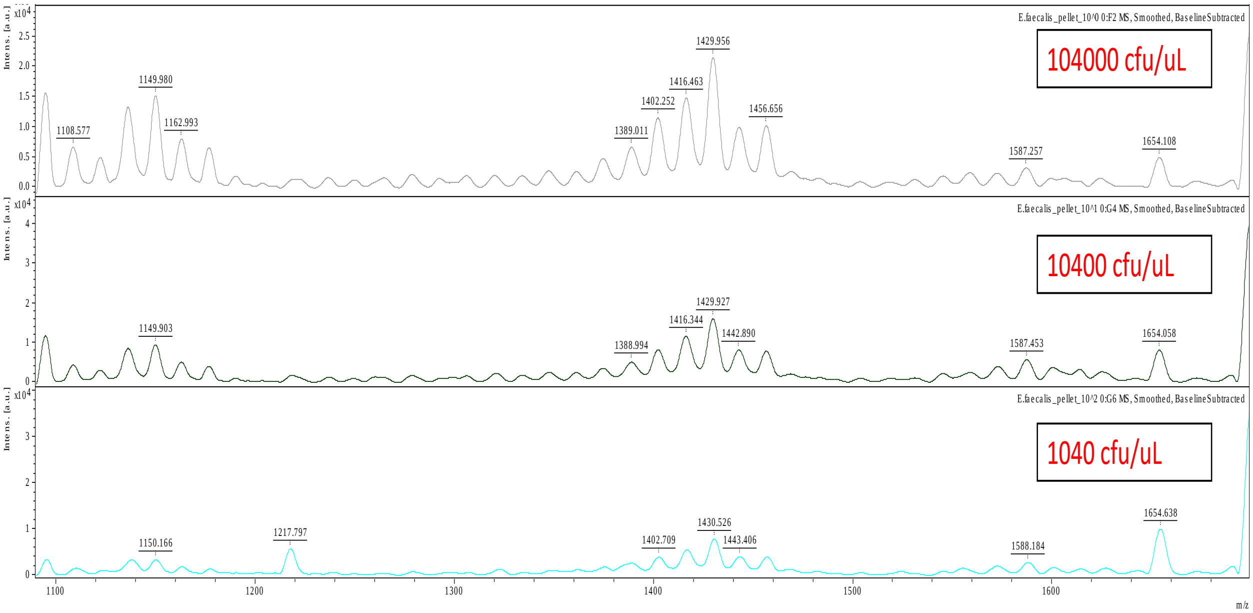

SI. Fig 3: Limit of detection of *E. faecalis*

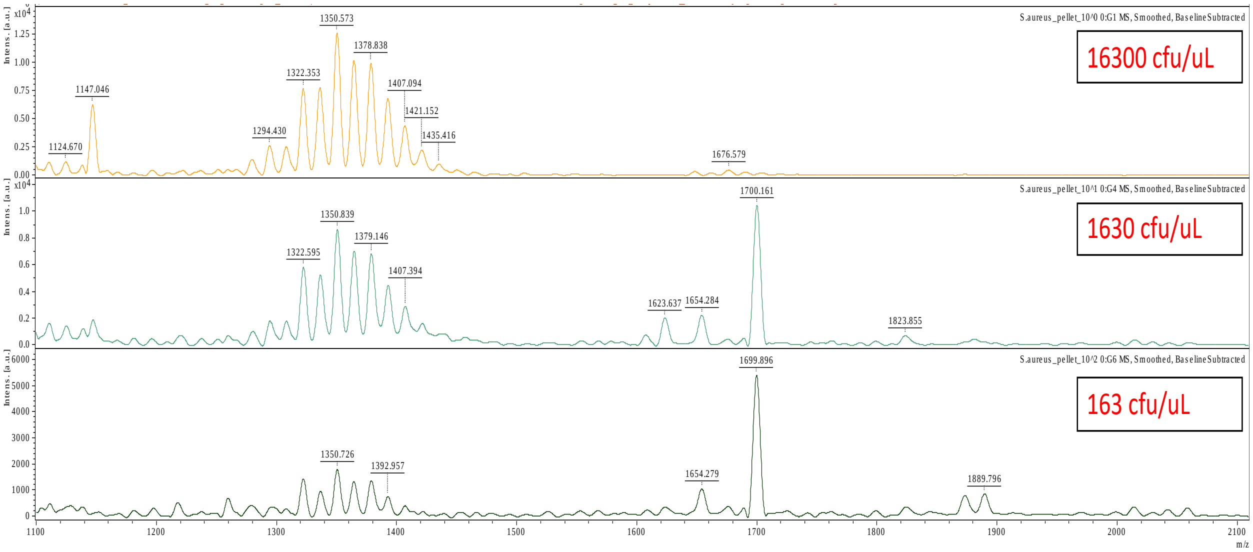

SI. Fig 4: Limit of detection of *S. aureus*

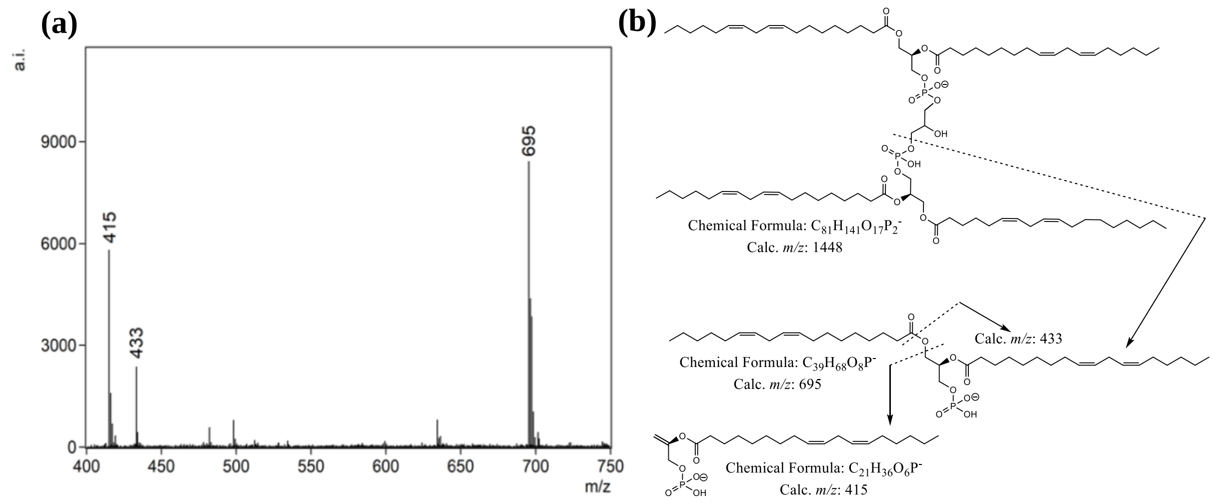

SI. Fig 5: FLAT mass spectrum of 1448 interferences (a) A mass spectrum of *m/z* 1448 ion selected for tandem MS (b) Predicted structure of 1448 ion. All data from MALDI-timsTOF-MS.

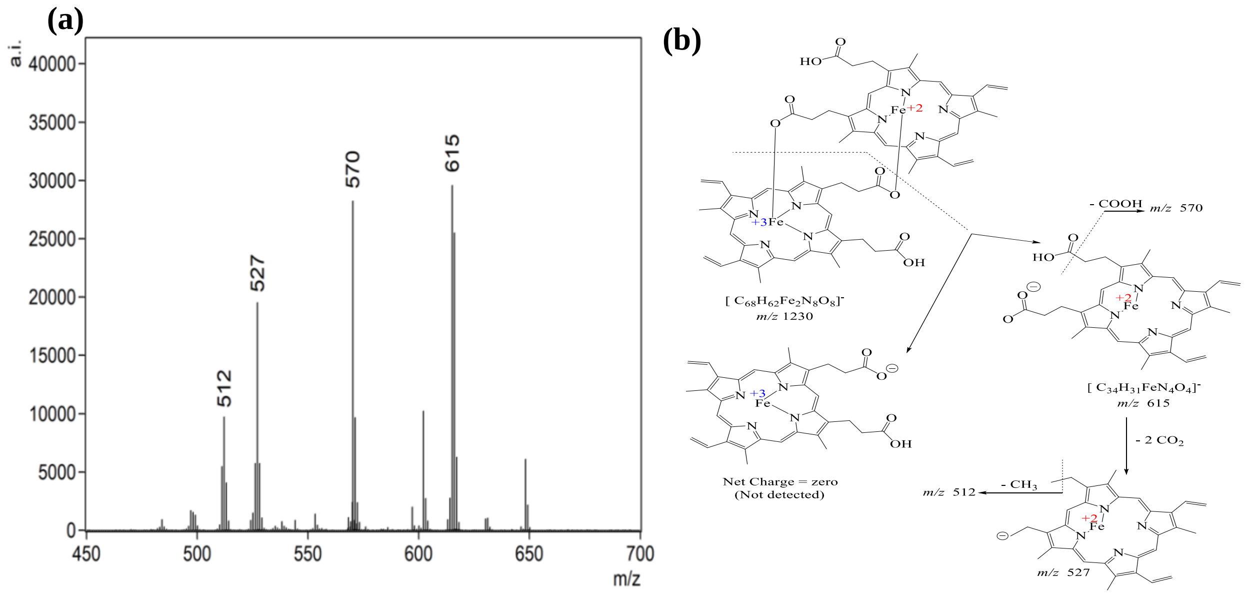

SI. Fig 6: FLAT mass spectrum of heme with the FLAT analysis of urine samples with blood: (a) Tandem MS spectra of 1230 ion (b) Predicted structure of 1230 ion. All data from MALDI-timsTOF-MS.
